# Integrated stress response genes as novel biomarkers and therapeutic targets in ankylosing spondylitis: a transcriptomic and Mendelian randomization study

**DOI:** 10.3389/fimmu.2026.1718471

**Published:** 2026-03-26

**Authors:** Shuai Wang, Jingliang Zhu, Honghui Zhang, Shengxuan Hu, Jiaxin Mei, Chusong Zhou, Benchao Shi

**Affiliations:** 1 Department of Spinal Surgery, Orthopedic Medical Center, Zhujiang Hospital, Southern Medical University, Guangzhou, China; 2 Department of Oncology, Zhujiang Hospital, Southern Medical University, Guangzhou, China; 3 Department of Orthopedic, Affiliated Hospital of Jiujiang University, Jiujiang, China; 4 The Second Clinical Medical College of Guangdong Medical University, Dongguan, China

**Keywords:** ankylosing spondylitis, biomarkers, integrated stress response, Mendelian randomization, *RORA*

## Abstract

**Introduction:**

Ankylosing spondylitis (AS) is a chronic inflammatory disorder with poorly defined pathogenic mechanisms. The integrated stress response (ISR), an evolutionarily conserved signaling network, is implicated in AS development. This research endeavored to identify biomarkers for AS, thereby offering novel targets and approaches for therapeutic intervention.

**Methods:**

Transcriptomic profiling of peripheral blood from AS patients identified differentially expressed genes. Mendelian randomization (MR) was applied to infer causal associations between ISR-related genes and AS susceptibility. Functional enrichment, immune infiltration, and drug prediction analyses were performed, followed by RT–qPCR validation of candidate biomarkers in clinically collected blood samples.

**Results:**

Both database analyses and clinical validation demonstrated marked downregulation of *RORA* and *FBXO31* and increased expression of *MSRB3* in AS. MR analysis substantiated their causal contributions to AS risk. Functional enrichment indicated involvement in olfactory transduction pathways, and strong correlations with immune infiltration, particularly Th1 cells and keratinocytes, were observed. Drug prediction suggested indirubin and pentoxifylline as potential therapeutic agents.

**Conclusion:**

The findings highlight ISR involvement in AS pathogenesis and identify novel biomarkers and therapeutic targets warranting further investigation.

## 1 Introduction

Ankylosing spondylitis (AS) is a chronic, progressive inflammatory disorder primarily involving the axial skeleton, characterized by inflammatory back pain that advances to spinal ankylosis, irreversible structural lesions, and functional decline (1, 2). Its global prevalence is estimated at 0.07–0.32%, with a male-to-female ratio of approximately 2–3:1 (3). Although strong genetic associations have been identified, particularly with HLA-B27, the precise etiology and pathogenesis remain unresolved. Current therapeutic strategies, including nonsteroidal anti-inflammatory drugs and biologics targeting TNF-α or IL-17, alleviate symptoms and delay progression but do not achieve a cure. In addition, several targeted approaches show limited benefit in AS; for instance, inhibition of the IL-23 pathway, while effective in related conditions, failed to produce clinical improvement in AS (4). Such therapeutic constraints highlight the imperative for defining novel molecular biomarkers and mechanistic targets to enable more precise and effective intervention strategies.

The integrated stress response (ISR) represents a conserved cytoprotective signaling program engaged under diverse cellular stress conditions. Four stress-sensing kinases—PERK, GCN2, PKR, and HRI—detect stimuli such as unfolded protein accumulation, nutrient deprivation, viral infection, and oxidative stress, converging on phosphorylation of eIF2α at Ser51. This modification suppresses global protein synthesis while enabling preferential translation of stress-adaptive mRNAs, including ATF4 (5). ISR activity initially promotes restoration of homeostasis; however, under persistent or severe stress, the pathway shifts toward apoptosis induction (6). Persistent or dysregulated ISR activation has been documented in multiple neurodegenerative disorders, notably Alzheimer’s and Parkinson’s diseases, although its specific contribution to AS pathogenesis remains unclear and requires further clarification.

Mendelian randomization (MR), which uses genetic variation to infer causal links between risk factors and disease outcomes (7), has recently been adopted in AS studies to identify etiological determinants and potential therapeutic targets (8).

In this study, the GEO and GWAS databases were utilized to identify ISR-related genes in AS through an integrated MR analysis of transcriptomic data, thereby revealing potential therapeutic targets. Validation was conducted using RT–qPCR on clinical blood specimens. In addition, GSEA and immune infiltration analysis were applied to investigate underlying regulatory pathways and candidate drugs, offering theoretical support for elucidating gene expression regulation, clarifying disease mechanisms, and informing novel drug development.

## 2 Methods

### 2.1 Data collection

AS-related datasets were retrieved from the GEO database (https://www.ncbi.nlm.nih.gov/geo/). The GSE18781 dataset (GPL570 platform) was designated as the training cohort and consisted of 18 AS peripheral blood samples and 25 controls. For validation, the GSE25101 dataset (GPL6947 platform) was employed, comprising 16 AS whole blood samples and 16 matched controls. In addition, a total of 989 ISR-related genes (IRGs) were obtained by merging 47 unfolded protein response genes, 79 heat shock response genes, 585 oxidative stress response genes, 119 hypoxia response genes, and 231 DNA damage response genes (9–11), (**Supplementary Table 1**).

Moreover, MR data associated with AS were obtained from the IEU Open GWAS database (https://gwas.mrcieu.ac.uk/). The AS dataset (ukb-b-18194) included 462,933 European participants, including 1,296 cases and 461,637 controls, with genotypic information on 9,851,867 SNPs. Expression quantitative trait locus (eQTL) data corresponding to exposure factors were also extracted from the IEU Open GWAS database.

### 2.2 Data analysis

#### 2.2.1 Differential expression analysis

In GSE18781, differentially expressed genes (DEGs) between AS and control groups were identified using “limma” (v 3.54.0) (12), with the threshold set at |log_2_ FC| > 0.5 and P < 0.05. The distribution of DEGs was displayed in a volcano plot generated with “ggplot2” (v 3.3.5) (13). A heatmap depicting the top 10 upregulated and downregulated genes was produced by “pheatmap” (v 1.0.12) (14)], providing a concentrated view of the most pronounced expression alterations.

#### 2.2.2 Determination and function analysis of candidate genes

Candidate genes were obtained by intersecting DEGs and IRGs through “VennDiagram” (v 1.7.1) (15). Functional enrichment analysis of the resulting genes was performed with “clusterProfiler” (v 4.6.0) (16), including Gene Ontology (GO) and Kyoto Encyclopedia of Genes and Genomes (KEGG) assessments, to delineate the associated biological processes and signaling pathways (P < 0.05).

#### 2.2.3 Data preprocessing of MR study

The MR analysis was conducted using the “TwoSampleMR” package (v 0.6.4), considering candidate genes as exposures and AS as the outcome (17). The procedure adhered to the three core assumptions of conventional MR: (1) independence, requiring that IVs are unrelated to confounders; (2) relevance, indicating a direct association between IVs and the exposure; and (3) exclusion restriction, stipulating that IVs influence the outcome solely through the exposure without alternative pathways.

Initially, IVs were identified with the extract_instruments function (P = 5×10^-6^). SNPs in linkage disequilibrium were eliminated (clump = TRUE, r² = 0.001, kb = 10). Variants associated with the outcome were excluded, while those linked to exposures were retained. Effect alleles and effect sizes were subsequently aligned using the harmonise_data function. The strength of each SNP was then quantified by F-statistics, with SNPs showing F < 10 discarded. Only SNPs with F > 10 were preserved, and analyses required at least three SNPs to proceed.

#### 2.2.4 MR study, sensitivity analysis, and Steiger test

After the selection of IVs, five algorithms (IVW (18), MR Egger (19), Weighted median (20), Simple mode (21), and Weighted mode (20)) were applied in the MR analysis using the MR function, with IVW regarded as the primary approach. The threshold for inclusion in the MR study was set at P_IVW_ < 0.05. The robustness of these findings was further assessed using FDR correction for evaluation purposes only, not as a filtering criterion. To evaluate the robustness of association signals for significantly exposed factors, genomic inflation factors (λGC) derived from the UK Biobank (UKB) and GWAS datasets were compared. Quantile–quantile (QQ) plots of p-values for both datasets were generated through the qqplot function in the stats package (v 4.3.1) (22). In addition, faceted bar charts illustrating both raw and standardized λGC values were constructed using ggplot2 (v 3.3.5).

Subsequently, graphical assessments were performed: scatter plots were used to examine relationships between exposures and outcomes, forest plots to visualize the effect size (odds ratio or beta) of each individual instrumental variable (SNP) on the outcome, along with the overall combined effect, and funnel plots to evaluate the symmetry of causal estimates. Sensitivity analyses were then conducted to confirm the reliability of the MR results. These included heterogeneity testing (Cochran’s Q test with P > 0.05 and I² < 25%), horizontal pleiotropy testing (P > 0.05), and leave-one-out (LOO) analyses implemented with mr heterogeneity (23), mr pleiotropy test and the MR-PRESSO method (NbDistribution = 1,000) (24), and mr leaveoneout functions (25). Finally, the Steiger test was applied to assess the causal direction, with significance defined as correct causal direction = TRUE and P < 0.05. Genes demonstrating significant causal associations with AS that satisfied both sensitivity analyses and the Steiger test were identified as candidate biomarkers for subsequent investigations.

#### 2.2.5 Determination and analysis of biomarkers

Candidate biomarkers were assessed through gene expression analysis on the GSE18781 and GSE25101 datasets. Genes displaying significant differential expression between AS and control samples (Wilcoxon tests; adjusted P < 0.05) and consistent expression trends across both datasets were designated as potential biomarkers for AS. False discovery rate correction was performed using the Benjamini-Hochberg method. To distinguish AS from control groups, two separate backpropagation neural network classifiers were independently developed and evaluated on the GSE18781 and GSE25101 datasets, respectively, using the “neuralnet” package (v 1.44.2). Each model featured a single hidden layer with 10 neurons. Input features were standardized, and training applied a high convergence threshold (tolerance = 0.1) along with a cross-entropy loss function to prevent overfitting. Model performance for each respective dataset was evaluated using ROC analysis via the “pROC” package (v 1.18.0) (26), with an AUC above 0.7 indicating acceptable predictive capacity. Concurrently, the Bootstrap self-sampling method (with 1000 repeated samples) was employed to calculate the 95% confidence interval for the AUC, thereby assessing the model’s stability.

#### 2.2.6 Gene set enrichment analysis

The functional significance of the biomarkers was investigated by applying GSEA to the GSE18781 dataset. The reference set ‘c2.cp.kegg.v7.0.symbols.gmt’ was retrieved from MSigDB. Correlations between biomarkers and other genes were systematically calculated and ranked in descending order with the “psych” package (v 2.4.3). Enrichment analysis was then conducted with “clusterProfiler” (v 4.6.0), considering P < 0.05 and |NES| > 1 as thresholds of significance. This procedure identified biological processes and signaling pathways potentially modulated by the biomarkers under study.

#### 2.2.7 Immune infiltration analysis

Immune infiltration was assessed using the GSE18781 dataset. The xCell algorithm (v1.1.0) computed scores for 64 immune cell types (27). Differences between AS and control groups were evaluated with the Wilcoxon test, and cell types with significant alterations (adjusted P < 0.05) were selected for subsequent investigation. Correlation analysis was then conducted with the “psych” package (v2.4.3) to examine associations both among differentially infiltrating immune cells and between these cells and identified biomarkers (|cor| > 0.30, adjusted P < 0.05). False discovery rate correction was performed using the Benjamini-Hochberg method.

#### 2.2.8 Small ubiquitin-like modifier analysis

SUMOylation regulates diverse biological processes, including cell cycle progression, DNA replication and repair, signal transduction, and metabolic regulation (28). To characterize SUMO modification sites of the identified biomarkers, protein sequences corresponding to the biomarkers were obtained from the NCBI database. FASTA files were downloaded and analyzed using GPS-SUMO 2.0 (https://sumo.biocuckoo.cn/) to predict SUMO interaction motifs and consensus sites at the protein level.

#### 2.2.9 Regulatory network analysis

Biomarkers were used as the gene set, and the “RcisTarget” package (v 1.23.1) was applied to predict transcription factors (TFs) regulating these biomarkers and to delineate the potential regulatory network (29). Overrepresentation analysis of each motif within the biomarker set was conducted, and motifs with the highest NES values were annotated with the corresponding motif–TF relationships. Furthermore, the expression patterns of TFs associated with the enriched motifs were examined to clarify their relevance to biomarker regulation.

#### 2.2.10 Drug prediction

Samples from GSE18781 were stratified into high- and low-expression groups based on the median biomarker expression level. Differential expression analysis between the two groups was performed using the “limma” package (v 3.54.0) with cutoffs of |log_2_FC| > 0.5 and P < 0.05 (12). Resulting DEGs were visualized through volcano plots and heatmaps, highlighting the top 10 upregulated and downregulated genes with the “ggplot2” and “pheatmap” packages, respectively. These DEGs were subsequently queried in the Connectivity Map (CMAP) database to identify candidate drugs targeting the dysregulated genes. To further examine the connections between the predicted drugs and their downstream pathways, the top 10 drugs (defined by the highest number of associated genes) and their corresponding pathways were illustrated in a Sankey plot generated with the “ggalluvial” package (v 0.12.5) (30).

#### 2.2.11 Statistical analysis

Statistical analyses were performed in R (v 4.2.2). Differences between groups were assessed using the Wilcoxon test, and statistical significance was defined as P < 0.05.

### 2.3 Clinical specimens and RT–qPCR validation

Participants and ethics: Peripheral venous blood was collected from AS patients at Zhujiang Hospital, Southern Medical University (Guangzhou, China) before treatment initiation, and from healthy controls without autoimmune or inflammatory disorders. AS diagnosis followed the modified New York criteria. Written informed consent was obtained from all participants. Ethical approval was granted by the Ethics Committee of Zhujiang Hospital, Southern Medical University, and the study adhered to the Declaration of Helsinki.

Sample processing and RNA preparation: Fasting EDTA blood was processed within 2 hours of collection. Peripheral blood mononuclear cells were isolated by density-gradient centrifugation, washed with PBS, snap-frozen, and preserved at −80 °C. Total RNA was extracted with TRIzol reagent (Thermo Fisher Scientific) and reverse-transcribed into cDNA using a commercial kit according to the manufacturer’s protocol.

RT–qPCR: The genes *RORA*, *FBXO31*, and *MSRB3* were examined, with primer sequences listed in **Supplementary Table 2**. Amplification was performed with SYBR Green chemistry on a QuantStudio™ 6 Flex real-time PCR system (Applied Biosystems, US) under standard cycling parameters, followed by melt-curve analysis. Each sample was assayed in technical triplicate, and no-template controls were included on each plate. GAPDH was used as the internal reference. Relative expression levels were determined by the 2^−ΔΔCt^ method, with the median value of the control group as the calibrator.

## 3 Results

### 3.1 Functions and pathways of candidate genes were explored

**Figure 1:**
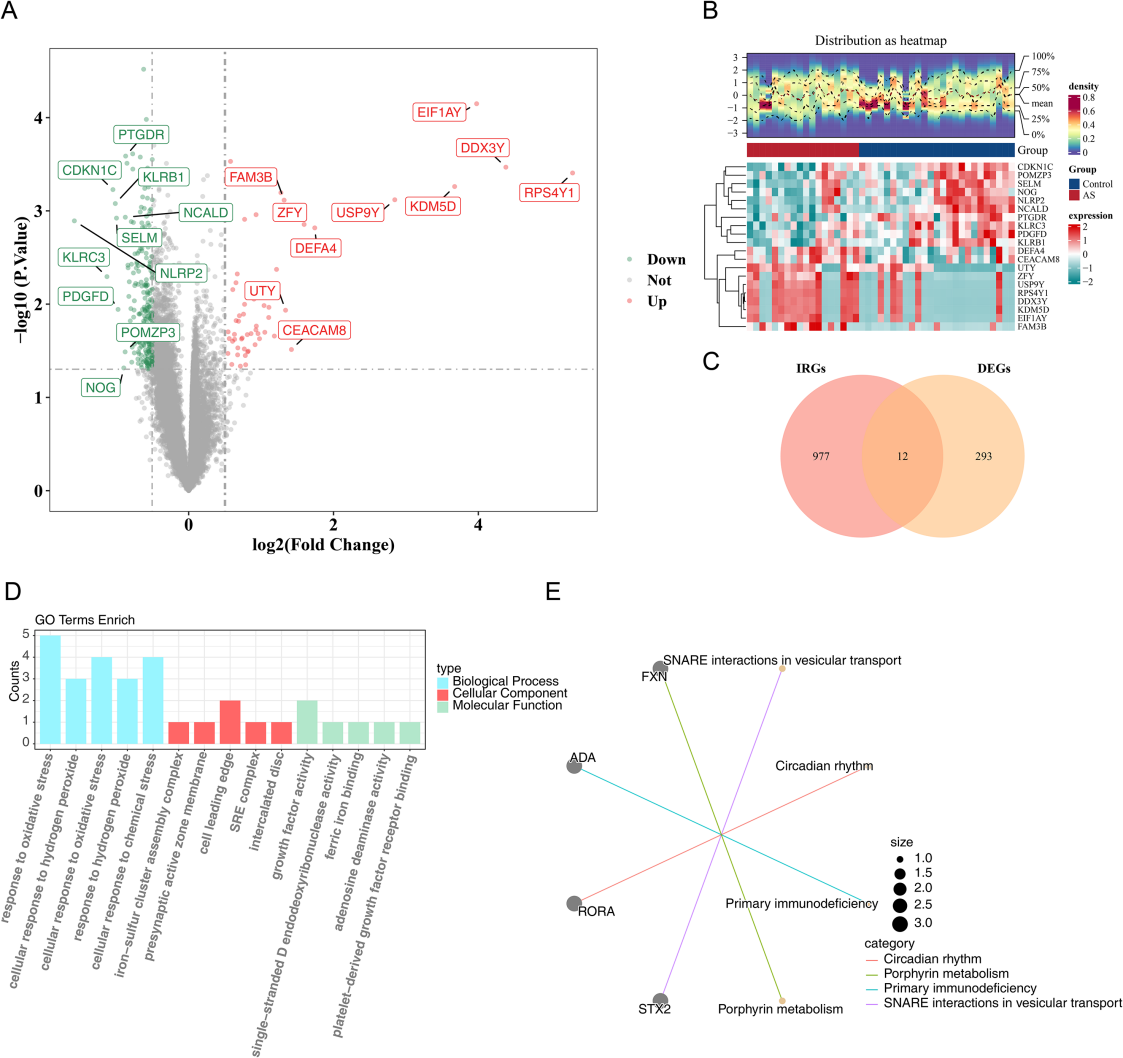
Determination of candidate genes. **(A)** Volcano plot of DEGs. Red dots represent the top ten up-regulated genes, and green dots represent the top ten down-regulated genes. **(B)** Heatmap of DEGs. Blue: control group; Red: AS group. **(C)** Venn diagram depicting 12 candidate genes. **(D)** GO enrichment of candidate genes. The x-axis indicates enriched pathways, the y-axis shows gene counts, and the right legend denotes BP, CC, and MF. **(E)** KEGG enrichment of candidate genes. Colored lines indicate enriched pathways, gray dots represent genes, connecting lines show gene–pathway associations, and dot size reflects enrichment significance.

A total of 305 DEGs were identified, including 56 up-regulated and 249 down-regulated genes in AS patients (**Figures 1A, B**). Cross-referencing these DEGs with 989 IRGs yielded 12 candidate genes (**Figure 1C**). Functional enrichment analysis revealed significant associations of the 12 genes with 403 GO terms, comprising 358 BPs, 10 CCs, and 35 MFs, along with 25 KEGG pathways (**Supplementary Table 3**). The most enriched GO terms included “response to oxidative stress” (BP), “cell leading edge” (CC), and “growth factor activity” (MF) (**Figure 1D**). The top four KEGG pathways were “circadian rhythm,” “porphyrin metabolism,” “primary immunodeficiency,” and “SNARE interactions in vesicular transport” (**Figure 1E**). Collectively, the candidate genes and enriched pathways appear to contribute to AS pathogenesis, particularly through mechanisms linked to immune regulation, oxidative stress, and metabolic processes.

### 3.2 Candidate biomarkers that showed a significant causal relationship with AS were ascertained

**Figure 2:**
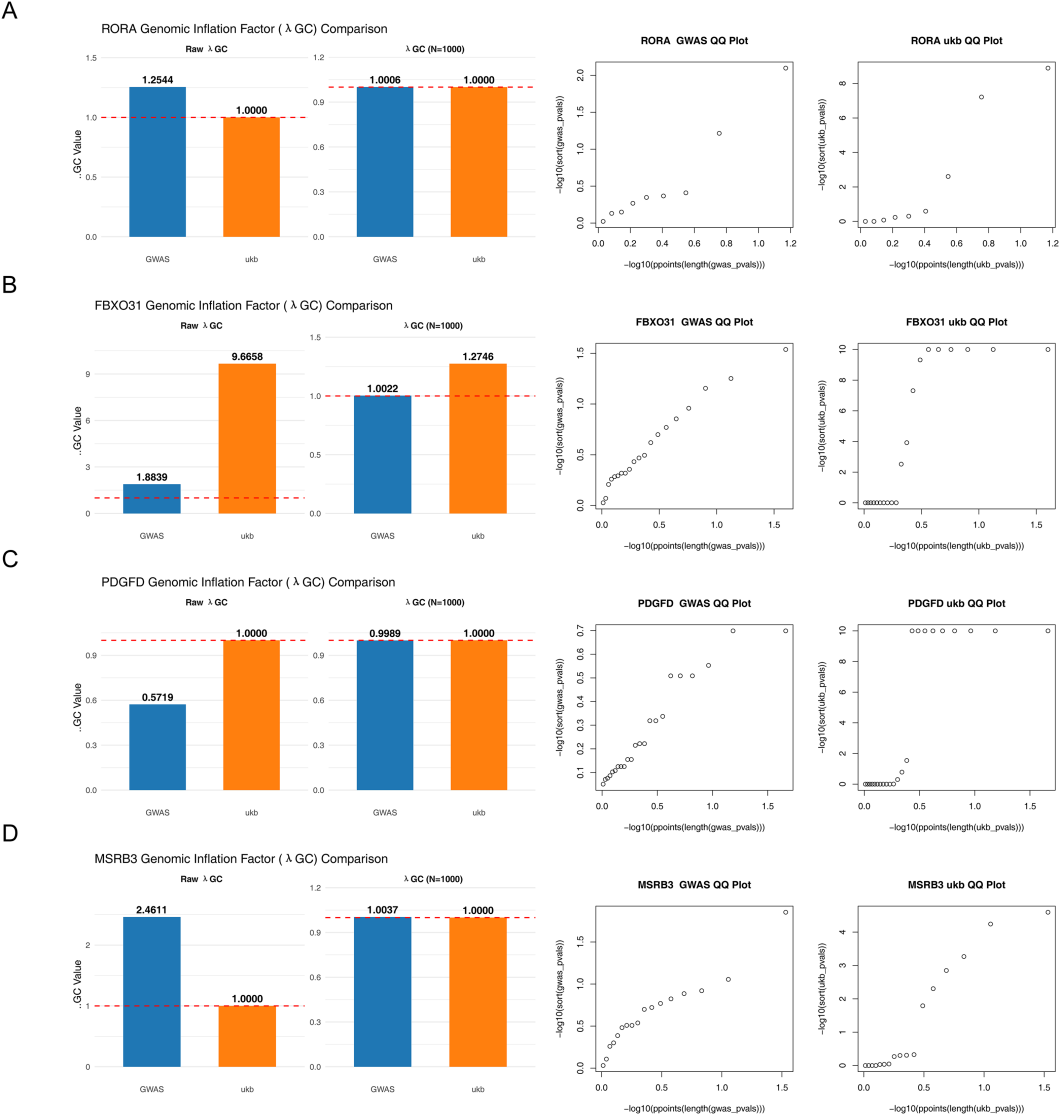
Results of Mendelian randomization analysis using Inverse Variance Weighted (IVW) method. **(A)** Q-Q plot and bar chart for *RORA*. **(B)** Q-Q plot and bar chart for *FBXO31*. **(C)** Q-Q plot and bar chart for PDGFD. **(D)** Q-Q plot and bar chart for *MSRB3*. Blue: GWAS; Orange: ukb. The x-axis represents theoretical −log10(P), and the y-axis represents observed −log10(P). Points clustered along the diagonal indicate agreement between observed and expected distributions (λGC ≈ 1).

From the 12 candidate genes, MR analysis identified four with significant causal relevance to AS (**Table 1**). All four demonstrated increased risk estimates [OR > 1, P < 0.05], with corresponding SNP F-values consistently exceeding 10 (**Supplementary Table 4**). The identified genes included *RORA* (P = 0.0493, OR = 1.0010, 95% CI = 1.0000–1.0021), *FBXO31* (P = 0.0009, OR = 1.0006, 95% CI = 1.0000–1.0009), PDGFD (P = 0.0035, OR = 1.0006, 95% CI = 1.0002–1.0011), and *MSRB3* (P = 0.0002, OR = 1.0016, 95% CI = 1.0007–1.0024). Yet, the FDR-adjusted P-value for RORA was 0.1791, suggesting that its causal relationship with AS requires further investigation. Q-Q plots and bar charts further confirmed the robustness of these associations. For *RORA*, λGC values were 1.0006 (GWAS) and 1.0000 (ukb) (**Figure 2A**); for *FBXO31*, 1.0022 (GWAS) and 1.2746 (ukb) (**Figure 2B**); for PDGFD, 0.9989 (GWAS) and 1.0000 (ukb) (**Figure 2C**); and for *MSRB3*, 1.0037 (GWAS) and 1.0000 (ukb) (**Figure 2D**). The λGC values for most tests were close to 1.0, indicating minimal inflation of false positives overall. The elevated value for FBXO31 (1.2746) suggested a subset of SNPs may represent authentic association signals, as reflected in the slight deviation at the extreme upper-right tail of the Q-Q plot.

**Table 1:** Mendelian randomization (IVW) results for candidate genes associated with AS risk.

id.exposure	Method	pval	pval-fdr	or
eqtl-a-ENSG00000069667 (RORA)	Inverse variance weighted	0.0493	0.1791	1.0010
eqtl-a-ENSG00000103264 (FBXO31)	Inverse variance weighted	0.0009	0.0101	1.0006
eqtl-a-ENSG00000170962 (PDGFD)	Inverse variance weighted	0.0035	0.0279	1.0006
eqtl-a-ENSG00000174099 (MSRB3)	Inverse variance weighted	0.0002	0.0094	1.0016

**Figure 3:**
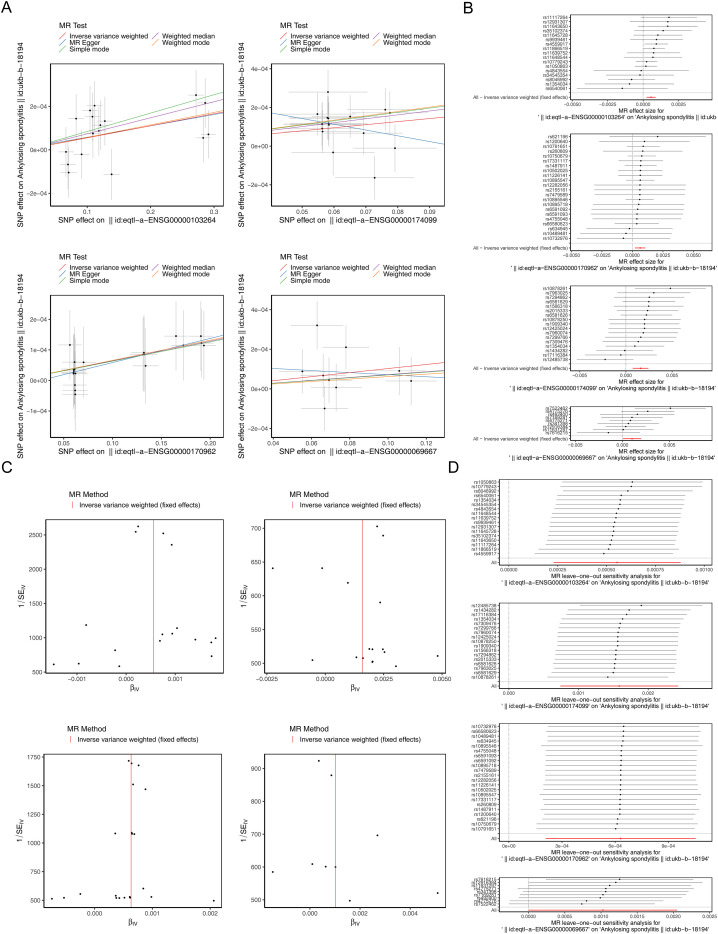
Verification of MR Results. **(A)** Scatter plots of MR analysis for four candidate biomarkers. Colored lines represent regression results from distinct MR algorithms. A positive slope suggests a risk factor, whereas a negative slope indicates a protective factor. A nonzero intercept implies confounding. **(B)** Forest plots of MR analysis for four candidate biomarkers. Solid lines entirely left of 0 suggest reduced risk with higher exposure, lines entirely right of 0 suggest increased risk, and lines crossing 0 indicate nonsignificant associations. **(C)** Funnel plots of MR analysis for four candidate biomarkers. The x-axis denotes the beta value of each IV, and the y-axis denotes the reciprocal of the standard error. **(D)** Forest plots of Leave-One-Out validation, where the x-axis shows SNP-specific effect estimates, the y-axis indicates SNP loci, and the red line marks the overall effect.

Scatter plots revealed consistent positive slopes across the four genes (**Figure 3A**). Forest plots displayed MR effect sizes, with IVW estimates exceeding 0 for each gene (**Figure 3B**). Funnel plots showed symmetrical distributions of individual SNP estimates around the IVW summary line, indicating no strong evidence of directional pleiotropy (**Figure 3C**). The heterogeneity test yielded P values above 0.05 for all genes (**Table 2**), and I² values below 25% indicated minimal heterogeneity (**Supplementary Table 5**). The horizontal pleiotropy test confirmed the absence of pleiotropy (P > 0.05) (**Table 3**; **Supplementary Table 6**). LOO analysis supported the stability of the MR estimates, as no marked deviations were observed (**Figure 3D**). The Steiger test further validated the correct causal direction for all four genes (P < 0.05), reinforcing the reliability of the MR results (**Supplementary Table 7**). Collectively, the four genes were identified as candidate biomarkers for subsequent research.

**Table 2:** The results of heterogeneity test.

id.exposure	Outcome	Method	Q_pval
eqtl-a-ENSG00000069667	ukb-b-18194	Inverse variance weighted	0.354454260881976
eqtl-a-ENSG00000103264	ukb-b-18194	Inverse variance weighted	0.60848478042203
eqtl-a-ENSG00000170962	ukb-b-18194	Inverse variance weighted	0.99999996736121
eqtl-a-ENSG00000174099	ukb-b-18194	Inverse variance weighted	0.658047656717678

**Table 3:** The results of horizontal pleiotropy test.

id.exposure	Outcome	P_val
eqtl-a-ENSG00000069667	ukb-b-18194	0.519504836192628
eqtl-a-ENSG00000103264	ukb-b-18194	0.916163313702886
eqtl-a-ENSG00000170962	ukb-b-18194	0.708561488559779
eqtl-a-ENSG00000174099	ukb-b-18194	0.190031312928986

### 3.3 *RORA*, *FBXO31*, and *MSRB3* were deemed as biomarkers for AS

**Figure 4:**
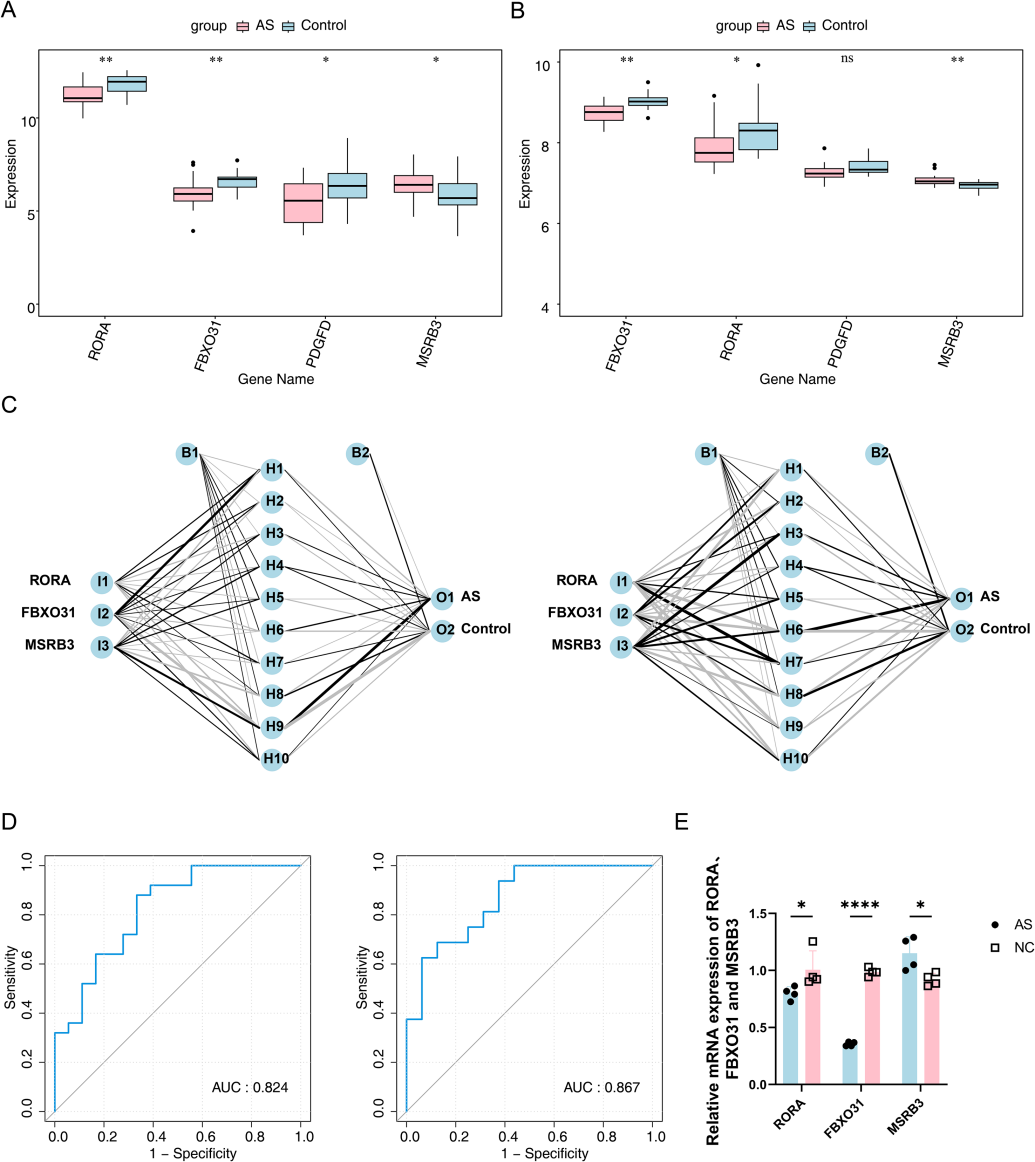
Determination of biomarkers. **(A)** Box plot of candidate biomarker expression levels in GSE18781. Red: AS group; Blue: Control group. **(B)** Box plot of candidate biomarker expression levels in GSE25101. Red: AS group; Blue: Control group. **(C)** Neural network constructed from biomarkers. Left: GSE18781; Right: GSE25101. Three genes serve as input nodes, the middle constitutes the hidden layer, and the right represents the output layer. Line thickness indicates weight magnitude; B1 and B2 denote optimization parameters. **(D)** ROC curves of neural networks. Left: GSE18781; Right: GSE25101. The x-axis indicates the false positive rate (fraction of negative samples incorrectly classified as positive), and the y-axis represents the true positive rate (fraction of positives correctly identified). **(E)** qPCR validation of the three biomarkers in blood samples from AS patients and healthy controls. Ns P≧0.05, * P<0.05, ** P<0.01,**** P<0.0001.

Expression profiling of the four candidate genes indicated significant downregulation of *RORA* and *FBXO31* in AS samples, whereas *MSRB3* exhibited consistently elevated expression in both the GSE18781 and GSE25101 datasets (adjusted P < 0.05) (**Figures 4A, B**). Consequently, *RORA*, *FBXO31*, and *MSRB3* were designated as biomarkers for subsequent analyses. Two BP neural network models were established based on these biomarkers (**Figure 4C**), each comprising three input nodes, a hidden layer with ten nodes, and two output nodes. ROC analysis demonstrated favorable predictive capacity, with AUC values of 0.824 (95% CI = 0.681–0.933) for GSE18781 and 0.867 (95% CI = 0.726–0.969) for GSE25101 (**Figure 4D**). Validation by RT–qPCR using peripheral blood samples from AS patients confirmed the database-based results: *RORA* and *FBXO31* were significantly downregulated, while *MSRB3* expression was elevated compared with healthy controls (**Figure 4E**). Collectively, the identified biomarkers provide a robust basis for further investigation in AS.

### 3.4 Pathways and immune cell infiltration of biomarkers were investigated

**Figure 5:**
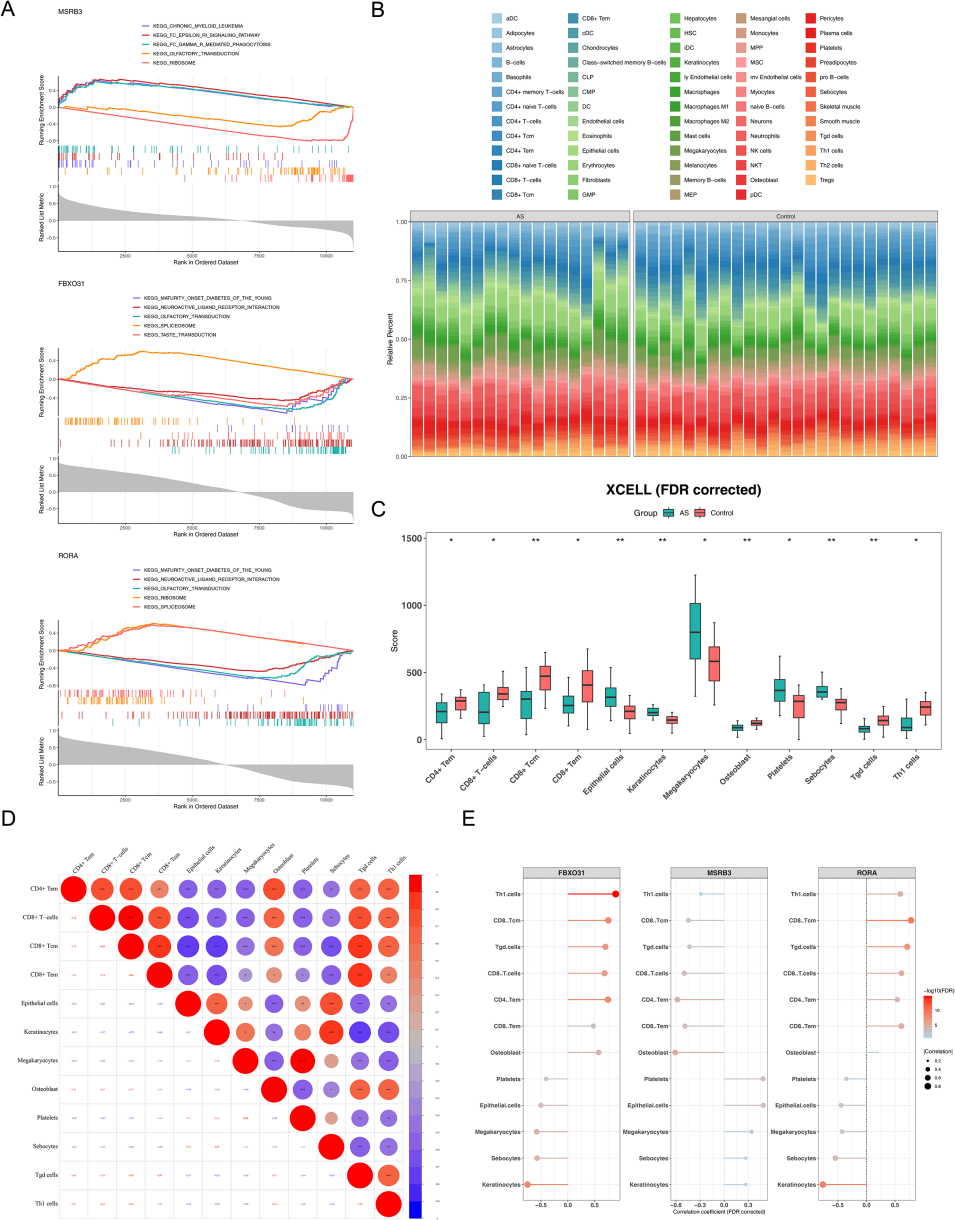
Pathways and immune cell infiltration of biomarkers. **(A)** Gene Set Enrichment Analysis of *RORA*, *FBXO31*, and *MSRB3*. The upper panel displays enrichment score curves, with each line corresponding to a pathway and the peak indicating the enrichment score. Genes preceding the peak constitute the core subset of that pathway. A peak positioned in the upper left reflects predominantly upregulated core genes in high- versus low-risk groups, whereas a peak in the lower right reflects predominantly downregulated core genes in the same comparison. The middle panel depicts the positions of genes within each gene set, and the lower panel shows the rank distribution of all genes. **(B)** Immune cell infiltration abundance plot, where distinct colors denote different immune cell types. **(C)** Violin plots illustrating variations in immune cell infiltration between high- and low-risk groups. Red denotes AS samples, blue denotes controls; **(D)** Correlation analysis between biomarkers and differential immune cells, with red indicating positive and blue indicating negative correlations. **(E)** Biomarker association analysis, where point size reflects the strength of correlation. * P<0.05, ** P<0.01,** *P<0.001.

GSEA identified significant enrichment of *RORA*, *FBXO31*, and *MSRB3* in 39, 36, and 55 pathways, respectively (**Supplementary Table 8**). *RORA* was mainly associated with pathways such as “ribosome,” “spliceosome,” and “olfactory transduction,” *FBXO31* with “spliceosome,” “taste transduction,” and “olfactory transduction,” and *MSRB3* with “ribosome,” “chronic myeloid leukemia,” and “olfactory transduction” (**Figure 5A**). The shared enrichment of all three biomarkers in the “olfactory transduction” pathway suggested this pathway might play a potential role in AS progression mediated by these biomarkers; however, this finding, based on our current computational analysis, requires further experimental validation. Immune cell infiltration analysis further revealed the profiles of 64 cell types in AS and control samples (**Figure 5B**). Wilcoxon test identified significant differences in 12 immune cell populations between AS patients and controls (adjusted P < 0.05) (**Figure 5C**). Keratinocytes and sebocytes were more abundant in AS tissues, whereas CD4+ memory T cells displayed higher infiltration in controls. Clarifying the biological contributions of these differential immune cells may advance understanding of AS pathogenesis and highlight potential therapeutic avenues. Among the 12 altered cell types, CD8+ Tcm showed the strongest positive correlation with CD8+ T cells (cor = 0.93, adjusted P < 0.05), whereas keratinocytes and epithelial cells were inversely correlated with CD8+ Tcm (cor = -0.72, adjusted P < 0.05) (**Figure 5D**). Biomarker–immune cell correlation analysis demonstrated a strong positive relationship between *FBXO31* and T helper 1 (Th1) cells (cor = 0.891, adjusted P < 0.05), while *RORA* exhibited a marked negative correlation with keratinocytes (cor = -0.765, adjusted P < 0.05) (**Figure 5E**; **Supplementary Table 9**). Based on our computational analyses, the results suggested that *FBXO31* might contribute to Th1 cell regulation and *RORA* could influence keratinocyte dynamics, thereby potentially affecting immune responses in AS, though these insights require further experimental validation.

### 3.5 The underlying molecular mechanisms of biomarkers were investigated

**Figure 6:**
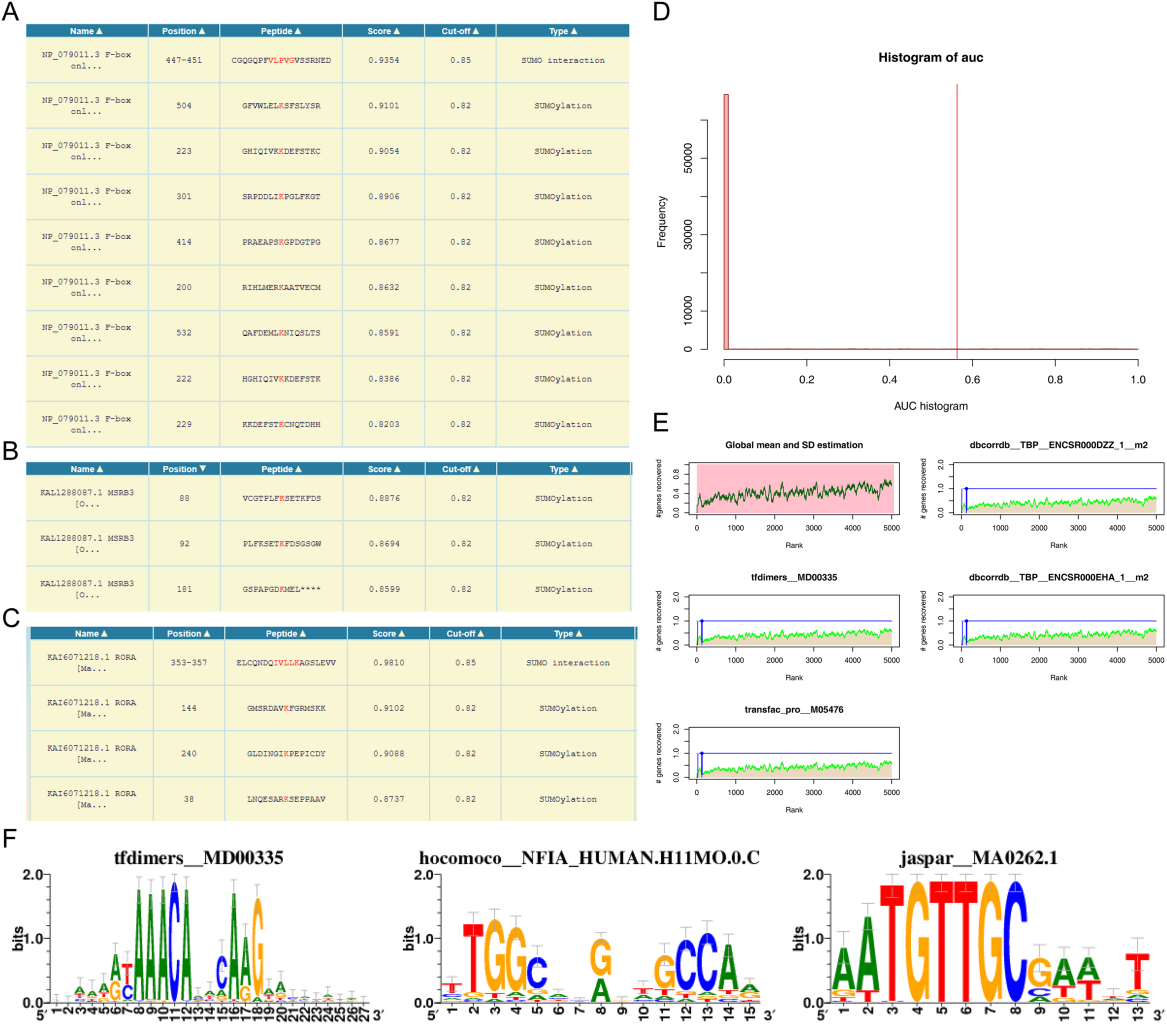
Potential molecular mechanisms of biomarkers. **(A–C)** Motifs with the optimal NES scores for *FBXO31*, *MSRB3*, and *RORA*. **(D)** TF annotation of motifs based on normalized enrichment score. A maximum enrichment score of 1 is considered enriched despite its relatively low value. **(E)** TF annotation based on normalized enrichment score, where the maximum distance point (mean+sd) from the green curve represents the highest enrichment level. **(F)** Motifs with optimal NES scores in key genes, specifically *FBXO31*, *MSRB3*, and *RORA*.

Analysis of SUMO-conjugation sites revealed one SUMO-interaction motif and eight consensus SUMOylation sites in *FBXO31* (**Figure 6A**). *MSRB3* contained three consensus SUMOylation sites (**Figure 6B**), whereas *RORA* harbored one SUMO-interaction motif and three consensus sites (**Figure 6C**). Cumulative recovery curves were subsequently applied to annotate motifs with their corresponding TFs. The enrichment profiles indicated that the top-ranked motif classes consistently belonged to class 1, reflecting strong enrichment (**Figures 6D, E**). *FBXO31*, *MSRB3*, and *RORA* exhibited marked enrichment in motifs including tfdimers MD00335, Hocomoco NFIA HUMAN.H11MO.0.C, and Jaspar MA0262.1 (**Figure 6F**). Collectively, the data delineate the involvement of these biomarkers in regulatory networks governed by specific motif–TF interactions.

### 3.6 Potential drug and targeted pathways of AS were mined

**Figure 7:**
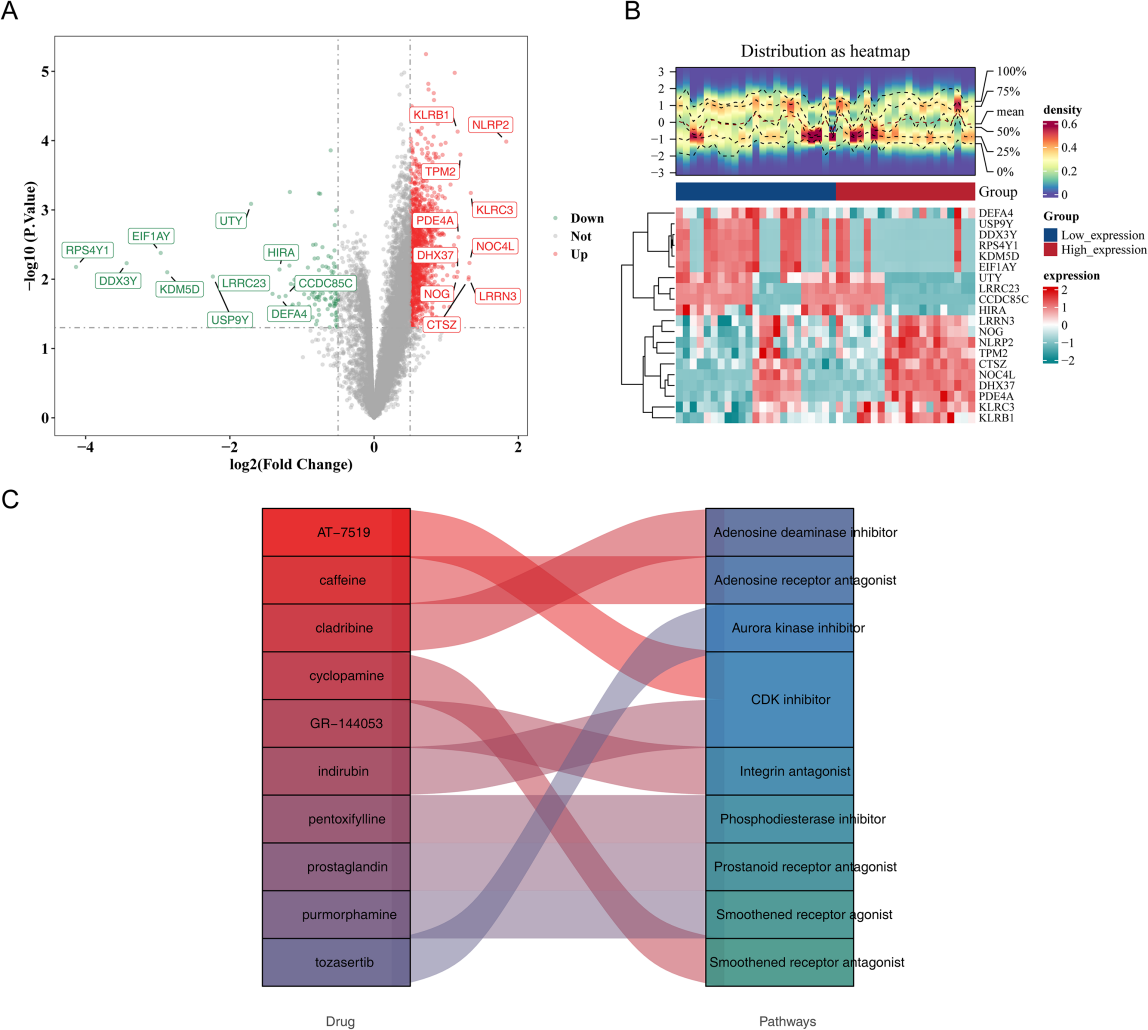
Potential drug and targeted pathways of AS. **(A)** Volcano plot of DEGs in high- versus low-expression groups of key genes. Red dots indicate the top 10 upregulated genes, and green dots indicate the top 10 downregulated genes. **(B)** Heatmap of DEGs in high- versus low-expression groups of key genes. The upper panel displays the gene density distribution in each sample, and the lower panel shows fold-change differences between groups, ranking the top 10 upregulated and downregulated genes by log2FC. **(C)** Sankey diagram illustrating the top 10 drugs and their corresponding pathways.

A total of 443 DEGs were identified between the high- and low-expression groups, comprising 401 upregulated and 42 downregulated genes in the high-expression cohort (**Figures 7A, B**). Based on this gene set, the top 10 candidate drugs were predicted: AT-7519, caffeine, cladribine, cyclopamine, GR-144053, indirubin, pentoxifylline, prostaglandin, purmorphamine, and tozasertib (**Figure 7C**). Pathway analysis further revealed the pharmacological targets of these compounds. For example, cladribine was associated with inhibition of adenosine deaminase, whereas cyclopamine was linked to antagonism of the smoothened receptor (**Figure 7C**). Collectively, the predicted drug–pathway interactions suggest potential therapeutic strategies for AS.

## 4 Discussion

AS is a chronic inflammatory disease predominantly involving the axial skeleton, with pathogenesis strongly linked to immune dysregulation in which the IL-23/IL-17 axis exerts a central influence (2). Accumulating evidence indicates that cellular stress responses, particularly the ISR, shape immune regulation in persistent inflammatory states (31). Transcriptomic profiling combined with MR identified three ISR-related biomarkers (*RORA*, *FBXO31*, *MSRB3*), with collective enrichment in the olfactory transduction pathway. This observation reveals novel connections between stress signaling and AS pathogenesis, suggesting potential therapeutic opportunities.

As an essential transcription factor for Th17 cell differentiation and functional maintenance, *RORα* promotes the expression of *IL-17A*, *IL-23R*, and *CCR6*, and participates in mitochondrial metabolism and energy homeostasis, thereby sustaining the pro-inflammatory activity of Th17 cells (32). In AS, the IL-17 signaling pathway is recognized as a central driver of chronic inflammation and aberrant bone remodeling, with RORα acting as an upstream regulator within this cascade (2). Experimental studies have demonstrated that selective inhibition of RORα significantly reduces Th17 cell differentiation and IL-17–associated cytokine production without impairing thymic T-cell development (32). Collectively, these findings indicate that RORα plays a key role in the immune regulation of AS, and that therapeutic targeting of RORα may enable more precise and safer modulation of the IL-23/IL-17 axis, offering a promising direction for the immunotherapy of AS.

Studies have shown that oxidative stress and mitochondrial dysfunction play important roles in the pathogenesis of AS. Mesenchymal stem cells (MSCs) derived from AS patients often exhibit decreased mitochondrial membrane potential, impaired oxidative phosphorylation (OXPHOS), and excessive accumulation of reactive oxygen species (ROS), leading to cellular senescence and reduced immunomodulatory capacity, thereby contributing to chronic inflammation (33). MSRB3, a mitochondria-localized oxidoreductase, maintains cellular redox homeostasis by repairing oxidized methionine residues and scavenging ROS (34). Recent studies have demonstrated that MSRB3 enhances OXPHOS activity, preserves mitochondrial function, and suppresses lipid peroxidation and ferroptosis under hypoxic or stress conditions, whereas its downregulation—such as through METTL3-mediated m6A modification—can exacerbate mitochondrial damage and cell death (34). Given that AS patients typically exhibit elevated oxidative stress and mitochondrial metabolic disturbances, MSRB3 may serve as a critical regulatory factor in this process. Restoration or enhancement of MSRB3 activity may reduce ROS levels, improve mitochondrial metabolism, and maintain the immunoregulatory capacity of MSCs, thereby attenuating inflammatory responses and potentially restoring Th17/Treg balance (35). Therefore, MSRB3 represents a promising therapeutic target in AS, and pharmacological strategies that upregulate its expression or activity may alleviate oxidative damage, improve mitochondrial function, and modulate immune responses, providing new insights for precision therapy in AS.

AS is characterized by chronic inflammation and abnormal bone formation, with ubiquitination and deubiquitination processes playing key roles in bone metabolism (36). Recent studies have shown that FBXO31, an E3 ubiquitin ligase component, negatively regulates osteogenic differentiation of human bone marrow–derived mesenchymal stem cells by promoting β-catenin ubiquitination and degradation (37). ince activation of the Wnt/β-catenin pathway promotes osteoblast differentiation and bone formation, FBXO31-mediated β-catenin degradation may contribute to the disrupted bone remodeling seen in AS (38). Moreover, the deubiquitinating enzyme USP53 can counteract FBXO31 activity and stabilize β-catenin to enhance osteogenesis (37). These findings suggest that FBXO31 may be involved in the pathological ossification of AS and could represent a potential therapeutic target. Inhibiting FBXO31 activity or blocking its interaction with β-catenin may help restore bone homeostasis in AS, though further validation in disease models is needed.

Co-enrichment of *RORA*, *FBXO31*, and *MSRB3* within the olfactory transduction pathway was also identified. Although conventionally associated with odor perception, olfactory receptors (ORs) have been increasingly recognized for their roles in immune regulation. ORs expressed on immune cells such as macrophages can modulate inflammatory activity; for instance, OR6A2 activation induces inflammasome assembly and IL-1β release, intensifying inflammatory responses (39). Similarly, Olfr78 responds to lactate, driving the formation of tumor-associated macrophages that suppress antitumor immunity (39). Clinically, diminished olfactory function is frequently noted in patients with autoimmune or chronic inflammatory disorders (40), suggesting a potential interface between systemic inflammation and olfactory signaling. It is therefore plausible that OR-mediated mechanisms contribute to AS by altering immune cell activity, particularly through modulation of cytokine secretion and migratory behavior, thereby influencing disease progression.

Immune infiltration analysis indicated potential functional relevance of the identified biomarkers in AS. *FBXO31* expression exhibited a strong positive correlation with Th1 cells, while *RORA* displayed an inverse association with keratinocytes, aligning with established aspects of AS immunopathology. Elevated Th1 cells producing IFN-γ are characteristic of chronic inflammation in AS (41). The results suggest that *FBXO31* may contribute to a Th1-dominant inflammatory milieu, potentially by strengthening immune cell adaptation to persistent inflammatory stress or by eliminating inhibitors of Th1 differentiation (42, 43). Although this interpretation remains tentative, *FBXO31* emerges as a putative regulator of Th1-driven inflammation in AS and warrants further experimental investigation.

Recent findings suggest that epidermal dysregulation may contribute to systemic immune activation in AS, extending beyond its traditional musculoskeletal pathology. In our study, RORA expression showed a significant negative correlation with keratinocyte abundance (cor = –0.765, P < 0.05), implying that aberrant keratinocyte activity may accompany decreased RORA signaling in AS. This is consistent with previous experimental evidence showing that epidermal RORα is a key transcriptional regulator of keratinocyte late differentiation and lipid metabolism, essential for maintaining barrier integrity and preventing inflammatory responses (44). Loss of RORα function impairs cornified envelope protein expression (loricrin, filaggrin) and alters ceramide composition, leading to barrier disruption and exaggerated cutaneous inflammation in animal models (44). Given that keratinocytes are increasingly recognized as active immunomodulatory cells capable of producing IL-1, IL-6, and TNF-α in chronic inflammatory conditions such as psoriasis and spondyloarthritis (45). impaired RORA-driven barrier homeostasis may facilitate antigen exposure and cytokine-driven immune activation in AS. Thus, RORA downregulation could bridge epidermal barrier dysfunction and systemic inflammation, providing a plausible link between epithelial stress responses and autoimmune activation. From a therapeutic perspective, targeting RORA signaling in keratinocytes may restore epidermal integrity and mitigate inflammatory amplification, highlighting RORA as a potential therapeutic node in the epithelial–immune axis of AS.

Through drug prediction analysis, we identified ten candidate compounds that may exert potential effects on AS through specific molecular pathways.

Cyclopamine, a Hedgehog pathway inhibitor, has been shown to attenuate inflammation and pathological ossification in autoimmune arthritis by suppressing TNF-α, IL-1β, and IL-6 expression, thereby protecting cartilage integrity (46). In AS, aberrant activation of the Hedgehog pathway promotes endochondral ossification, and elevated serum Indian hedgehog (Ihh) levels that decline following anti-TNF therapy highlight its key role in inflammation-induced new bone formation (47, 48).

Indirubin, a bisindole compound derived from Indigo naturalis, has demonstrated the ability to inhibit Jak3/STAT3 signaling and suppress IL-1β, IL-6, IL-23, and IL-17 expression, thereby limiting Th17-mediated inflammation (49, 50). Given that the IL-23/IL-17 axis is central to AS pathogenesis, indirubin or its derivatives may have potential as modulators of related immune pathways, but direct evidence in AS remains limited.

Pentoxifylline, a phosphodiesterase inhibitor that decreases TNF-α and IL-1β production, has shown mild anti-inflammatory benefits in patients with rheumatoid arthritis (51, 52). These findings suggest possible applicability in spondyloarthritis, but supporting data in AS are still preliminary.

Other compounds, such as Cladribine, Caffeine, and AT-7519, may also exert potential effects; however, current evidence remains limited, and further experimental and clinical studies are required to clarify their therapeutic relevance.

This study established a multi-layered framework linking ISR-related genes to AS by integrating transcriptomic profiling with MR analysis. Three candidate biomarkers—*RORA*, *FBXO31*, and *MSRB3*—were identified as differentially expressed in AS and potentially causally associated with disease susceptibility, and their expression patterns were further validated in clinical blood samples using RT–qPCR. Functional interrogation through pathway enrichment highlighted the olfactory transduction pathway as a key mechanistic axis bridging ISR signaling with immune dysregulation, while immune infiltration analysis delineated the cellular context of these alterations. In parallel, candidate therapeutics targeting the identified dysregulated networks were prioritized. Collectively, the results provide a stress-response–oriented perspective on AS pathogenesis, yielding verifiable biomarkers and therapeutic targets for subsequent investigation. This work indicates that AS extends beyond a strictly immune-mediated condition, involving cellular stress responses and noncanonical signaling pathways, thereby opening new avenues for mechanistic study and therapeutic development.

Notably, we acknowledge an apparent discrepancy wherein our MR analysis suggested a positive association between genetically predicted expression of RORA/FBXO31 and AS risk (OR>1), while their observed expression was downregulated in AS case samples. This observation does not necessarily invalidate the MR inference. The MR result reflects a lifelong, genetically determined causal effect based on instrumental variables (eQTLs). In contrast, the measured downregulation in patients represents the transcriptional state within the established disease milieu, which may be influenced by compensatory feedback, medication, or other disease-related physiological changes. Therefore, a therapeutic strategy aimed at upregulating these genes seeks to correct their disease-associated deficiency, moving expression toward a homeostatic range, rather than contradicting the MR-implicated causal pathway. This distinction between etiological risk and disease-state expression is important for interpreting integrative genomic findings.

While this study provides novel insights, several limitations must be acknowledged. First, our conclusions are derived primarily from integrated bioinformatics and MR analyses, supplemented by qPCR validation in a relatively small clinical cohort, which limits the statistical power and generalizability of the findings. Second, detailed treatment histories were unavailable for the public GEO cohorts and incompletely captured in our clinical samples; thus, we cannot fully exclude the possibility that the observed expression signatures are influenced by pharmacodynamic effects rather than purely reflecting disease etiology. Third, although standard sensitivity analyses were performed, the inherent limitations of two-sample MR—including potential residual confounding, weak instrument bias, and horizontal pleiotropy—may still affect the causal interpretations. Finally, the current evidence remains at the transcriptional level without direct functional validation. Future studies employing larger, treatment-naive prospective cohorts, protein-level assays, and experimental models are needed to definitively establish the biological roles of RORA, FBXO31, and MSRB3 in AS. Notwithstanding these limitations, the consistent implication of the olfactory transduction pathway across our analyses suggests it may represent a functionally relevant nexus between cellular stress responses and immune dysregulation in AS, warranting further mechanistic investigation.

## 5 Conclusion

Transcriptomics-guided MR analysis identified FBXO31 and MSRB3 as putatively causal biomarkers for AS risk within the ISR network, while RORA emerged as a potential key biomarker primarily validated through consistent differential expression across cohorts.

GSEA of these biomarkers indicated shared enrichment in pathways such as “olfactory transduction,” implying potential involvement in disease pathogenesis. Immune infiltration profiling revealed 22 immune cell subsets with significantly altered abundance between AS and controls, and their associations with identified biomarkers were systematically examined. Regulatory network construction combined with drug prediction further highlighted potential therapeutic agents for AS. Together, the integrated results offer new perspectives for biomarker-based diagnosis and therapeutic development in AS.

## Data Availability

The datasets used during the current study are available in the NCBI GEO repository, including GSE18781 and GSE25101. The analysis scripts for this study are publicly available in Github at: https://github.com/fypwangshuai-stack/CODE.

## Supplementary material

Supplementary Table 1 Summary table of integrated stress response-related genes.

Supplementary Table 2 Summary table of primers of RT-qPCR used in this study.

Supplementary Table 3 Summary table of GO analysis of candidate genes.

Supplementary Table 4 Summary table of correlation results between 4 genes and AS.

Supplementary Table 5 Summary table of heterogeneity testing in MR.

Supplementary Table 6 Summary table of MR level pleiotropy test.

Supplementary Table 7 Summary table of Steiger test for four biomarkers.

Supplementary Table 8 Summary Table of GSEA analysis for RORA, FBXO31, and MSRB3.

Supplementary Table 9 Summary Table of biomarker association analysis.

## Glossary

ISR: Integrated Stress Response
IRGs: ISR-Related Genes
AS: Ankylosing Spondylitis
MR: Mendelian Randomization
DEGs: Differentially Expressed Genes
Th1: T Helper 1
GWAS: Genome-Wide Association Study
eQTL: Expression Quantitative Trait Locus
LD: Linkage Disequilibrium
LOO: Leave-One-Out
SUMO: Small Ubiquitin-like Modifier
TFs: Transcription Factors
CMAP: Connectivity Map
