# Supplementary material for: Integrated stress response genes as novel biomarkers and therapeutic targets in ankylosing spondylitis: a transcriptomic and Mendelian randomization study
Source: Front Immunol. 2026 Mar 26;17:1718471. doi: 10.3389/fimmu.2026.1718471 (PMC13062218; doi:10.3389/fimmu.2026.1718471)
Supplement: Supplementary Table 2 — Summary table of primers of RT-qPCR used in this study. [file DataSheet2.docx]

| Gene | Primers forward(5’-3’) | Product size(bp) |
| --- | --- | --- |
| RORA | Forward:CAC CAG CAT CAG GCT TCT TTC TC | 23 |
|  | Reverse:GTA TTG GCA GGT TTC CAG ATG GG | 23 |
| FBXO31 | Forward:GAG TTC TCC ACC AAG TGC AAC C | 22 |
|  | Reverse:TCC TGC ATG TGC TCG TGG AAG A | 22 |
| MSRB3 | Forward:CTA TGG GAT GCA CAG GGT GGA A | 22 |
|  | Reverse:CGC AGG TGT AAA AGA CAA GGC AG | 23 |
| GAPDH | Forward:ACT CTA CCC ACG GCA AGT TC | 20 |
|  | Reverse:TGG GTT TCC CGT TGA TGA CC | 20 |

**Primers of RT-qPCR used in this study**
